# Supplementary figures and images for: Prevalence of metabolic dysfunction-associated fatty liver disease and its association with glycemic control in persons with type 2 diabetes in Africa: A systematic review and meta-analysis
Source: PLOS Glob Public Health. 2024 May 6;4(5):e0002835. doi: 10.1371/journal.pgph.0002835 (PMC11073701; doi:10.1371/journal.pgph.0002835)

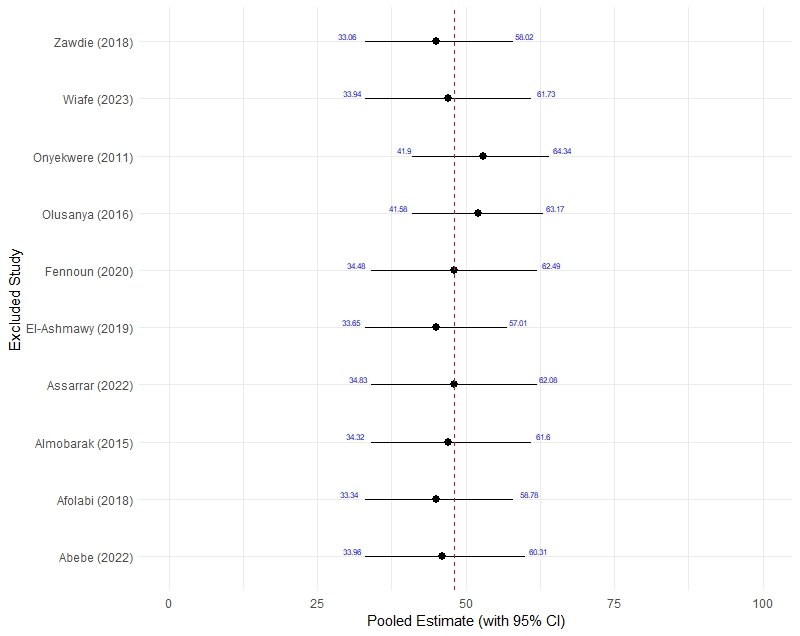


Sensitivity analysis of the effect of individual studies on the pooled prevalence of MAFLD

Supplement: S1 Fig — (DOCX) [file pgph.0002835.s002.docx]
