# Supplementary material for: Prevalence of metabolic dysfunction-associated fatty liver disease and its association with glycemic control in persons with type 2 diabetes in Africa: A systematic review and meta-analysis
Source: PLOS Glob Public Health. 2024 May 6;4(5):e0002835. doi: 10.1371/journal.pgph.0002835 (PMC11073701; doi:10.1371/journal.pgph.0002835)
Supplement: S2 Fig — (DOCX) [file pgph.0002835.s003.docx]

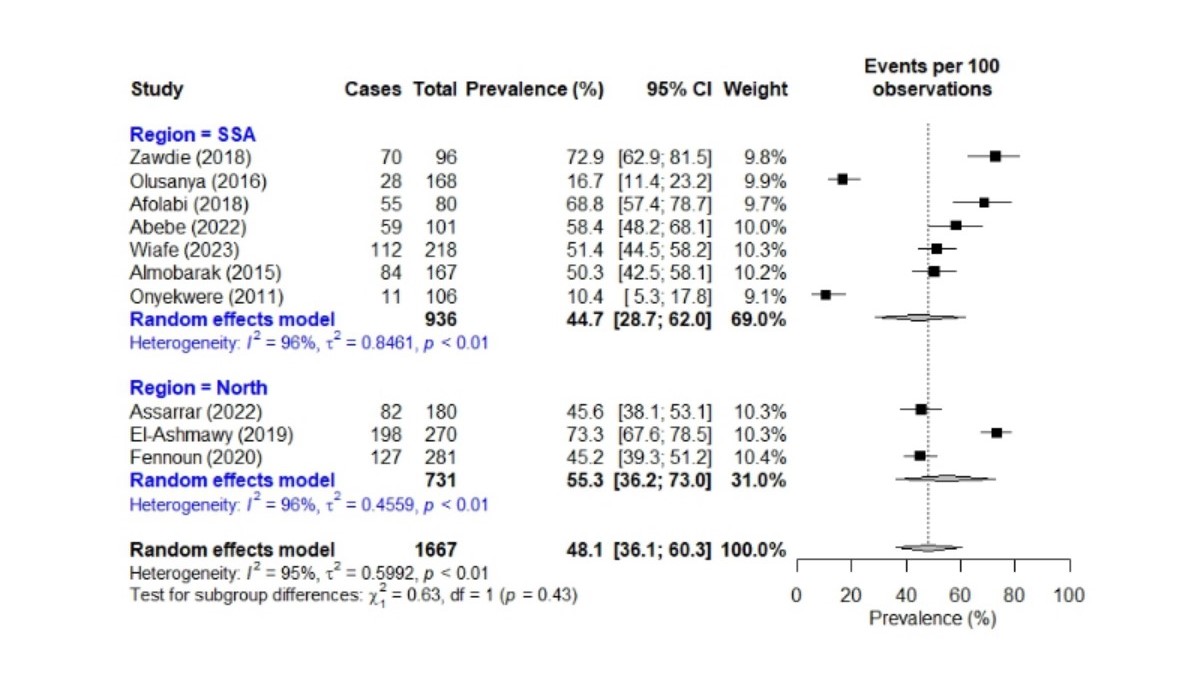


Forest plot of the subgroup analysis MAFLD prevalence based on region


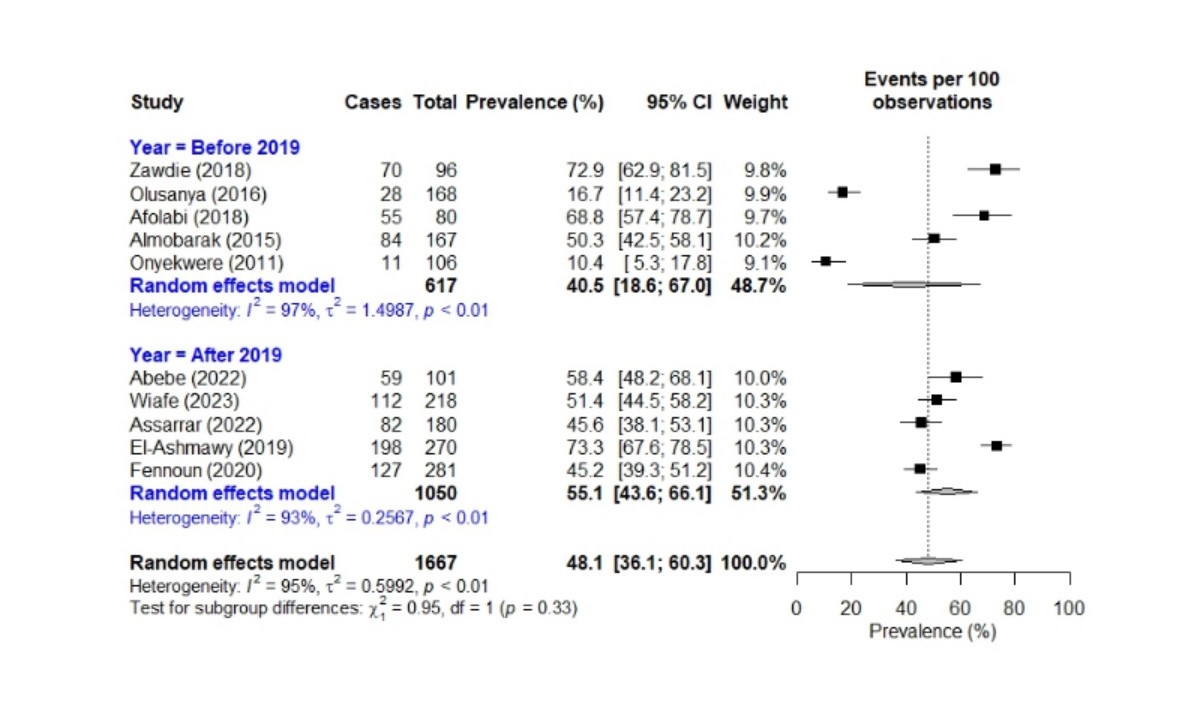


Forest plot of the subgroup analysis MAFLD prevalence based on year of study publication


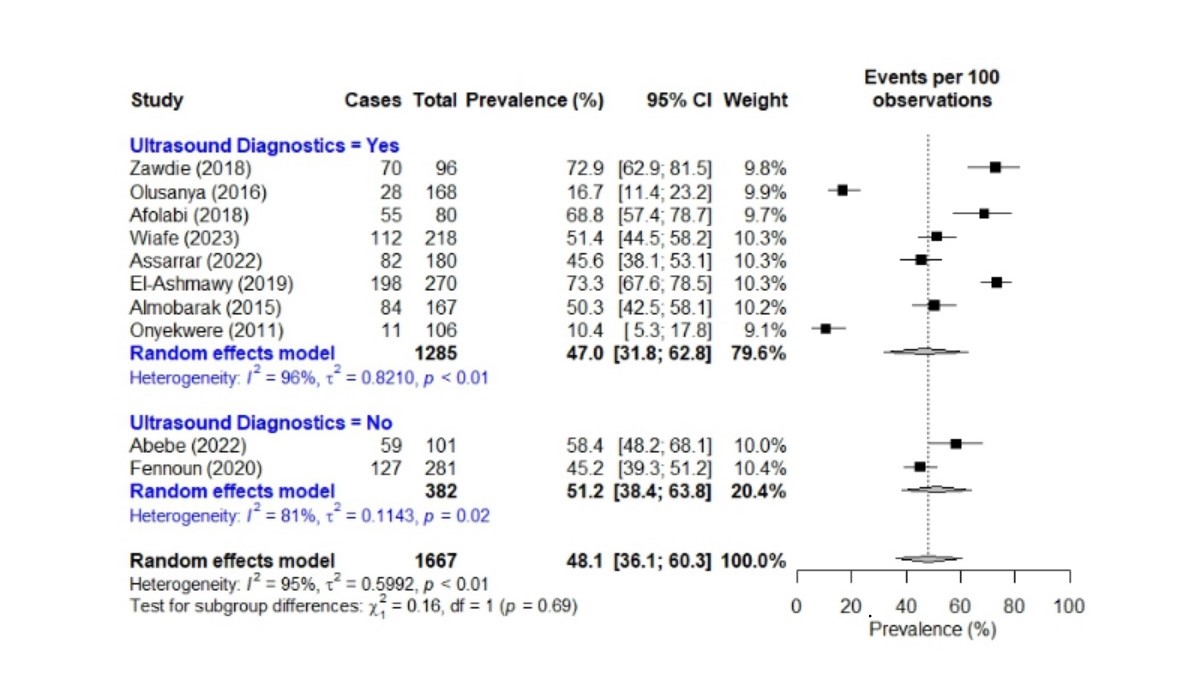


Forest plot of the subgroup analysis MAFLD prevalence based on diagnostic modality
